# What Is New in the Anti–*Pseudomonas aeruginosa* Clinical Development Pipeline Since the 2017 WHO Alert?

**DOI:** 10.3389/fcimb.2022.909731

**Published:** 2022-07-08

**Authors:** Sébastien Reig, Audrey Le Gouellec, Sophie Bleves

**Affiliations:** ^1^Laboratoire d’Ingénierie des Systèmes Macromoléculaires (LISM), Institut de Microbiologie, Bioénergies et Biotechnologie (IM2B), Aix-Marseille Université-CNRS, UMR7255, Marseille, France; ^2^Laboratoire Techniques de l’Ingénierie Médicale et de la Complexité (UMR5525), Centre National de la Recherche Scientifique, Université Grenoble Alpes, VetAgro Sup, Grenoble INP, CHU Grenoble Alpes, Grenoble, France

**Keywords:** *Pseudomonas aeruginosa*, multi-drug resistance, development pipeline, vaccine, *immunotherapy*, antibiotics, phage therapy, anti-virulence strategy

## Abstract

The spread of antibiotic-resistant bacteria poses a substantial threat to morbidity and mortality worldwide. Carbapenem-resistant *Pseudomonas aeruginosa* (CRPA) are considered “critical-priority” bacteria by the World Health Organization (WHO) since 2017 taking into account criteria such as patient mortality, global burden disease, and worldwide trend of multi-drug resistance (MDR). Indeed *P. aeruginosa* can be particularly difficult to eliminate from patients due to its combinatory antibiotic resistance, multifactorial virulence, and ability to over-adapt in a dynamic way. Research is active, but the course to a validated efficacy of a new treatment is still long and uncertain. What is new in the anti–*P. aeruginosa* clinical development pipeline since the 2017 WHO alert? This review focuses on new solutions for *P. aeruginosa* infections that are in active clinical development, i.e., currently being tested in humans and may be approved for patients in the coming years. Among 18 drugs of interest in December 2021 anti–*P. aeruginosa* development pipeline described here, only one new combination of β-lactam/β-lactamase inhibitor is in phase III trial. Derivatives of existing antibiotics considered as “traditional agents” are over-represented. Diverse “non-traditional agents” including bacteriophages, iron mimetic/chelator, and anti-virulence factors are significantly represented but unfortunately still in early clinical stages. Despite decade of efforts, there is no vaccine currently in clinical development to prevent *P. aeruginosa* infections. Studying pipeline anti–*P. aeruginosa* since 2017 up to now shows how to provide a new treatment for patients can be a difficult task. Given the process duration, the clinical pipeline remains unsatisfactory leading best case to the approval of new antibacterial drugs that treat CRPA in several years. Beyond investment needed to build a robust pipeline, the Community needs to reinvent medicine with new strategies of development to avoid the disaster. Among “non-traditional agents”, anti-virulence strategy may have the potential through novel and non-killing modes of action to reduce the selective pressure responsible of MDR.

## Introduction

As a worldwide public health threat, the World Health Organization (WHO) established a list of antibiotic-resistant bacteria in 2017 (Tacconelli et al., 2018). The aim was to prioritize and stimulate research and development strategies of new active drugs. Among relevant criteria such as patient mortality prevalence, health-care burden, and trend of resistance worldwide, carbapenem-resistant *Pseudomonas aeruginosa* (CRPA) were considered “critical-priority” bacteria (Tacconelli et al., 2018). Indeed, Carbapenem antibiotics are reserved for the treatment of multi-drug resistant (MDR) bacterial infections, and, when bacteria develop resistance to them, treatment options become extremely limited.

**Figure 1:**
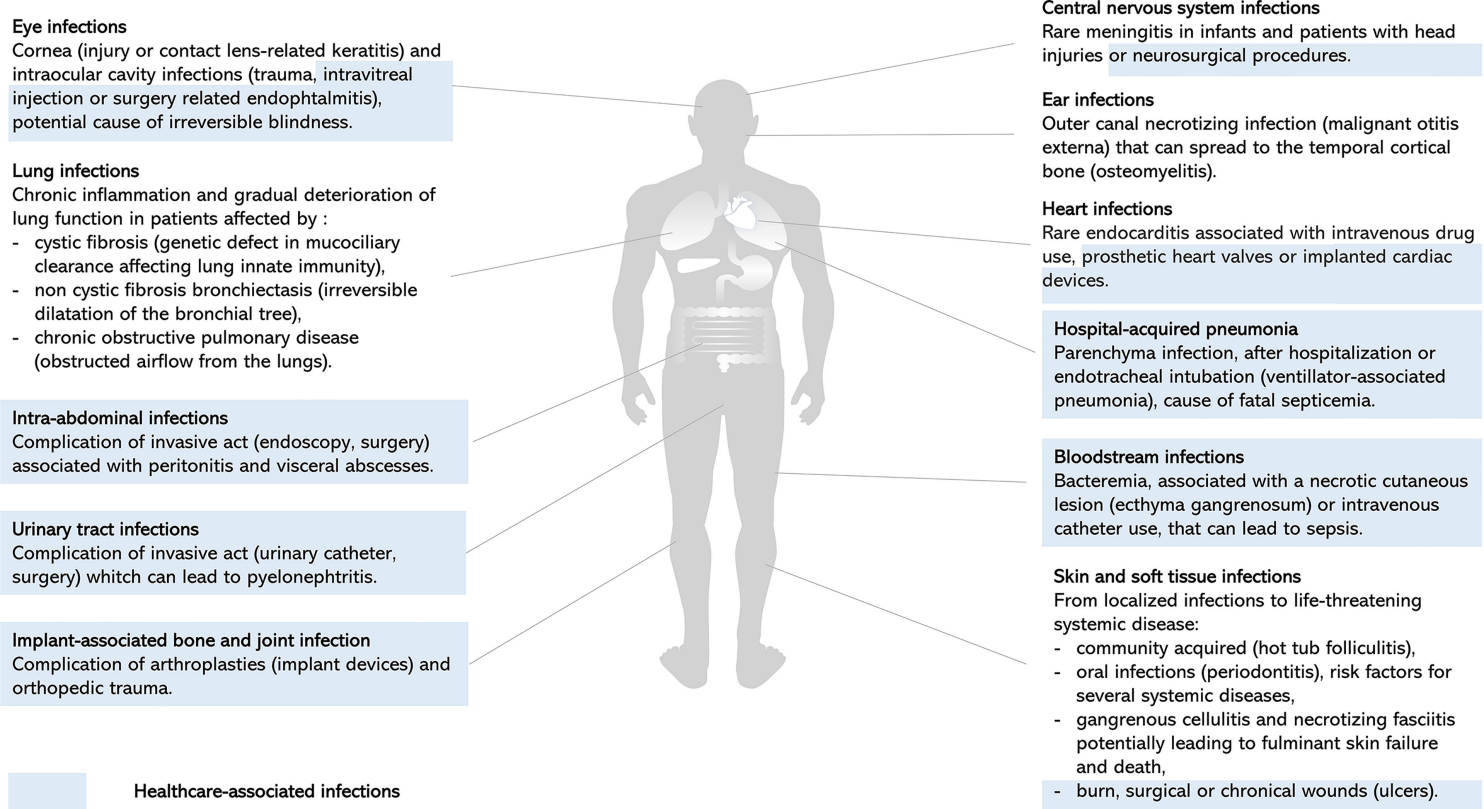
Clinical manifestations of *P. aeruginosa* infections. Representation of human body site infections and main clinical manifestations of *P. aeruginosa.* Healthcare-associated infections highlighted in blue illustrate the significant burden of *P. aeruginosa* on invasive acts, surgery, and device use, resulting in local or systemic complications (Wu et al., 2011; Dando et al., 2014; Gahlot et al., 2014; Elborn, 2016; Durand, 2017; Newman et al., 2017; Arsovic et al., 2020; Ramireddy et al., 2020; Chai and Xu, 2020; Shukla et al., 2020; Jean et al., 2020; Montravers et al., 2020; Cerioli et al., 2020; Shrestha et al., 2021; Vieira et al., 2016; Hauser and Ozer, 2011).

**Figure 2:**
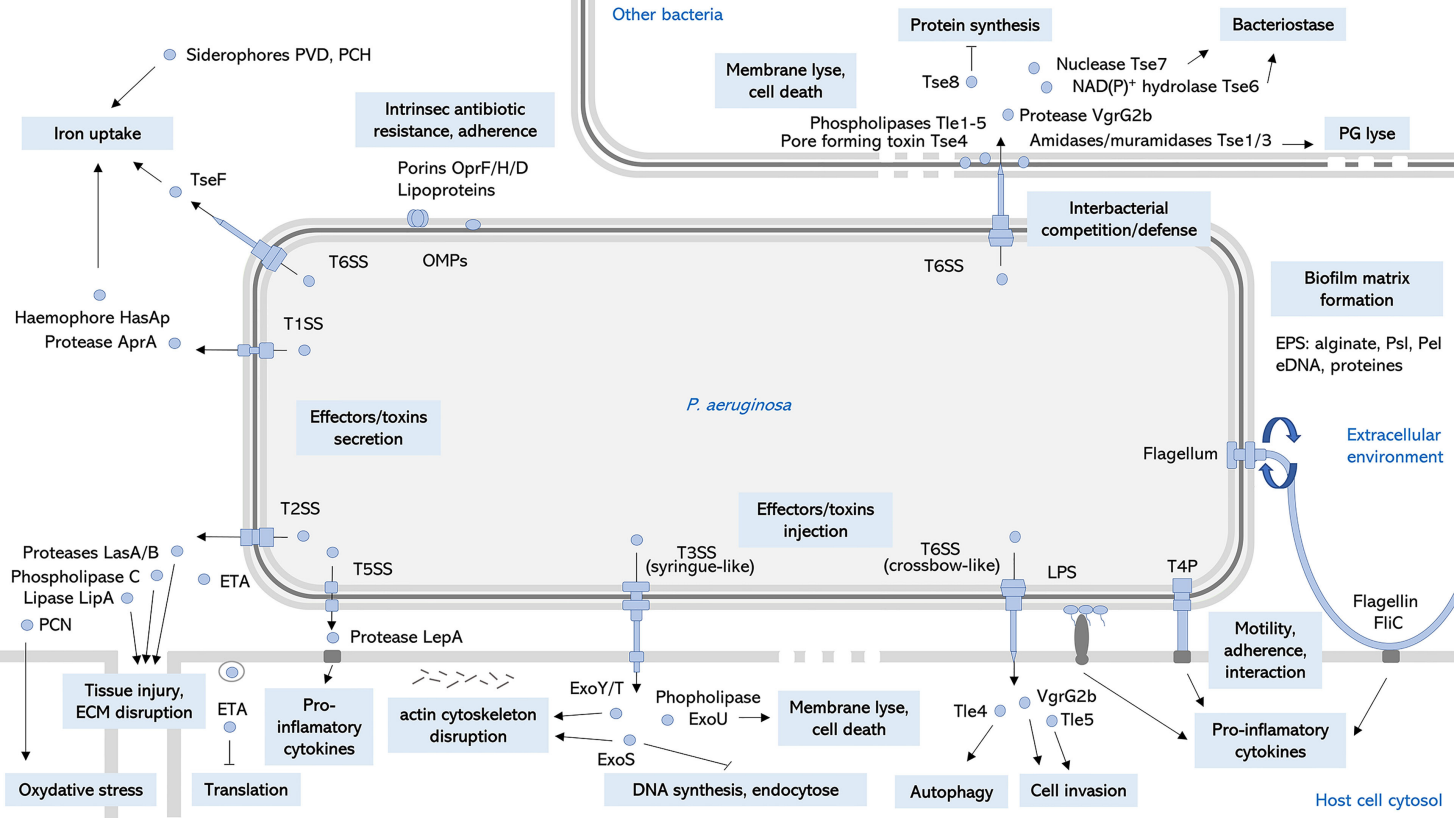
Key virulence factors of *P. aeruginosa. S*chematic representation of cell-associated and extracellular relevant virulence factors and their main roles on *P. aeruginosa* pathogenesis. OMPs, outer membrane proteins; LPS, lipopolysaccharide; ROS, reactive oxygen species; EPS, exopolysaccharides; eDNA, extracellular desoxyribonucleic acid; T4P, type 4 pili; TnSS, type n secretion system; ETA, exotoxin A; PVD, pyoverdine; PCH, pyochelin; PCN, pyocyanin; PG, peptidoglycan; ECM, extracellular matrix (Alhazmi, 2015; Sana et al., 2016; Berni et al., 2019; Jurado-Martín et al., 2021; Nolan et al., 2021).

*P. aeruginosa* is an opportunistic pathogen responsible for both severe acute and chronic infections, and is a significant cause of healthcare-associated infections, particularly in critically ill and immunocompromised patients (**Figure 1**). Since its first description in wound infections (Gessard, 1882), *P. aeruginosa* is now a well-known pathogen. Pathogenesis of *P. aeruginosa* is mediated by an arsenal of virulence factors: motility, adherence to biotic and abiotic surfaces, secreted toxins also called effectors that are released in the environment or injected into host cells or other bacteria (**Figure 2**). These effectors are able to modulate or disrupt host cells signaling pathways, target extracellular matrix, induce tissue damage, and shape the local microbiome by competition (**Figure 2**). The ability of *P. aeruginosa* to form a biofilm is also a key factor that increases drug resistance and escape from host defense and is responsible for colony tolerance to disinfectants on medical devices (Mulcahy et al., 2014; Pang et al., 2019; Jurado-Martín et al., 2021). As all these factors contribute to pathogenicity by complementary actions, *P. aeruginosa* is characterized by a combinatorial multifactorial virulence. Moreover, multiple mechanisms of antibiotic resistance have been identified, including intrinsic membrane permeability, drug efflux systems, production of antibiotic-inactivating enzymes, and loss of porin function (Pang et al., 2019). Finally, the plasticity of its (i) virulence factor gene expression, (ii) antibiotic resistance, and (iii) metabolism in response to selective pressure is one of the most challenging features of *P. aeruginosa* allowing the transition from acute to chronic infections. Acute infections are mainly associated with planktonic life style, whereas biofilm plays a major role in persistent infections. This remarkable ability of over-adaptation in a dynamic way allows this pathogen to escape immune system and become MDR or extensively drug resistant (XDR). Once a chronic infection in the patient is established, *P. aeruginosa* is really difficult to treat.

To meet this major treatment issue, research is active with a variety of approaches ranging from disarming the pathogen with an anti-virulence strategy to eliminating it. In parallel with the growing knowledge of the molecular mechanisms of *P. aeruginosa*–host interaction, the number of potential therapeutic targets is increasing. However, the path to validate efficacy of a new drug in human is long and uncertain. Two antibacterial tracks of development are followed and consist in “traditional agents” (small molecule directly targeting the bacterium for a bacteriostatic or bactericidal action) and new therapeutics called “non-traditional” (large molecule and/or not acting by directly targeting bacterial components essential for bacterial growth). Characterized by new, non-bactericidal, and generally species-specific modes of action, the paradigm is that non-traditional agents are less likely to generate the dreaded resistance (Tse et al., 2017). Indeed a non-killing agent reduces the selective pressure, and its specificity avoids cross-resistance potentially induced by horizontal genes transfer (Tse et al., 2017). This review focuses on new solutions for *P. aeruginosa* infections that are in active clinical development, i.e., currently being tested in humans and may be approved for patients in the coming years. A literature search was performed in a context of need to address clearly the question: What is new in the anti–*P. aeruginosa* clinical development pipeline since the 2017 WHO alert?

## Rationale of This Review and Methodology of Data Search

**Figure 3:**
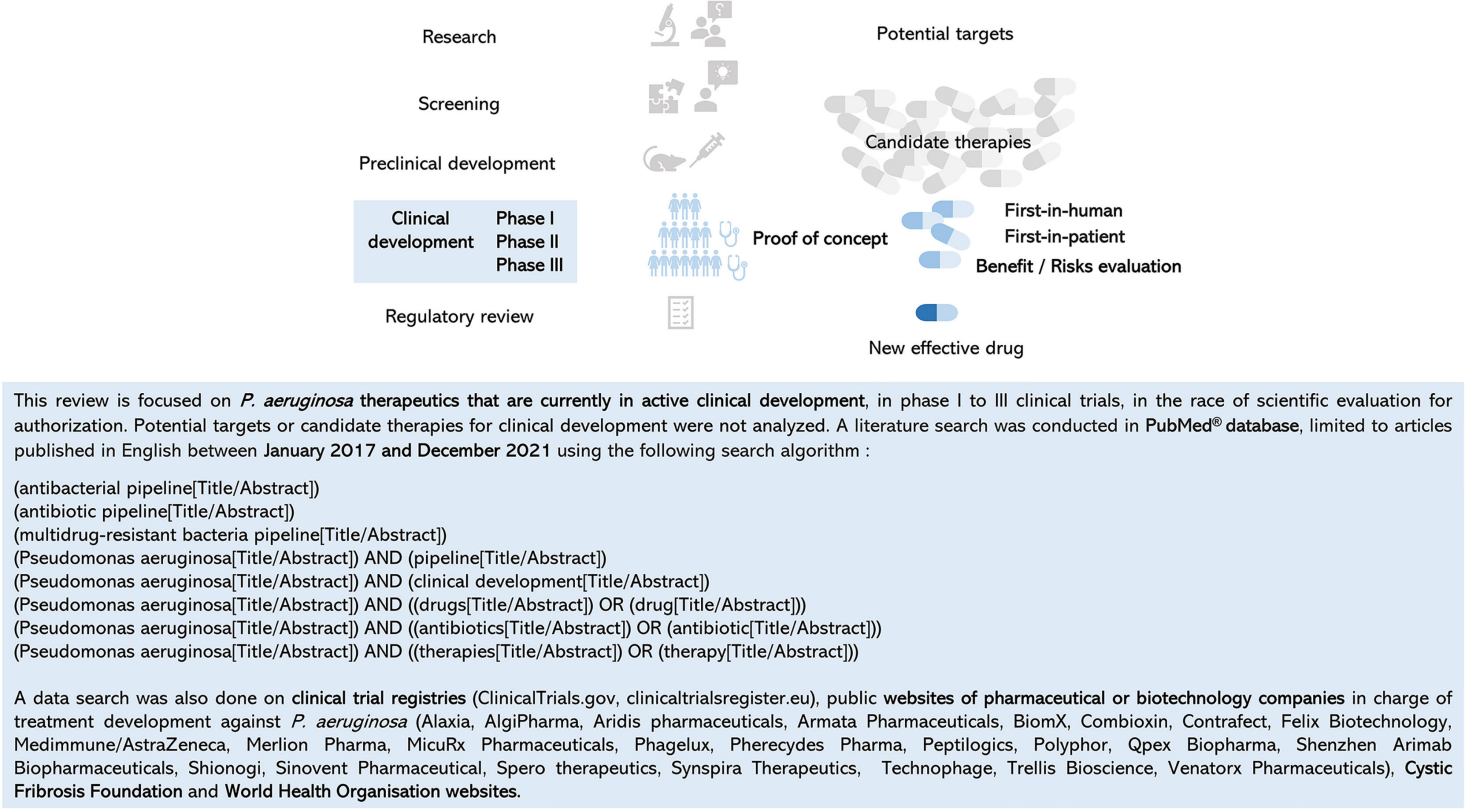
Review focus, data search criteria, and strategy.

With more than 74,000 articles published in PubMed^®^ in December 2021, knowledge about *P. aeruginosa* is growing in the fields of its virulence, antibiotic resistance, lifestyle, metabolism, clinical manifestations, clinical or observational studies, health and economic burden, among others. Numerous articles or reviews detail the potential new targets from basic research, the emerging new candidate therapies in early phase, or the pipeline of antibacterial molecules in preclinical and clinical testing and the hope that comes with it (Burrows, 2018; Tümmler, 2019; WHO, 2020; Theuretzbacher et al., 2020; WHO, 2021; Yaeger et al., 2021). It is difficult to find one’s way through all this data, which is often mixed and rarely dedicated to *P. aeruginosa*. It is important to keep in mind that before proof of concept in a phase II study, no efficacy is proven in patients. Efficacy–safety balance evaluation in *P. aeruginosa–*infected patients is an unavoidable milestone. To dedicate this review only to anti–*P. aeruginosa* treatments in clinical development, a literature search was conducted with a specific strategy in the PubMed^®^ database between January 2017 and December 2021. The following criteria were used: (i) keywords related to development (antibacterial pipeline, antibiotic pipeline, and clinical development) and treatment (drug, antibiotic, and therapy), (ii) selection of phase I to III clinical trials, and (iii) exclusion of research data or preclinical targets (**Figure 3**). Clinical trial registries and websites of companies or organizations with a special interest in the field were consulted to verify or supplement the data (**Figure 3).**

## Vaccines: A Prophylactic Strategy for High-Risk Patients

A vaccine against *P. aeruginosa* for at-risk patients [i.e., those older than 65 years and those with cystic fibrosis (CF), bronchiectasis, or chronic obstructive pulmonary disease] could reduce the prevalence of infections, the overall burden disease, and the use of antibiotic treatment. Therefore, although formal data are lacking, the vaccine, by limiting the use of antibiotics and thus reducing selection pressure on pathogens, has a significant indirect impact on the emergence of antibiotic resistance (López-Siles et al., 2021; Micoli et al., 2021). An important challenge for obtaining a good vaccine is the identification of ideal antigens, i.e., antigens accessible or presented to the immune system, adequately immunogenic and highly conserved across all serotypes (Sainz-Mejías et al., 2020), regardless of the stage or location of the infection. Antigen variability, plasticity of virulence factor expression during acute to chronic infection described earlier in the introduction, and bacterial localization (the mucosal immune response is compromised in dehydrated and sticky mucus in lungs of CF patients) are a challenge. In addition, it is also necessary to find a safe but immunogenic vector and antigen formulation (with or without adjuvant), allowing mucosal vaccination, intracellular and extracellular delivery of antigens to achieve specific cellular and humoral immunity, as well as long-term immunity. Finally, the choice of the target pathology is also very important: bacteremia, chronic lung infection, keratitis, urinary tract, or skin infection for the clinical trial.

Despite active research for a *P. aeruginosa* vaccine during over half a century, no vaccine has yet been approved (Sainz-Mejías et al., 2020; López-Siles et al., 2021; Micoli et al., 2021). Several antigens [lipopolysaccharide (LPS), alginate, flagellum, type 4 pili, outer membrane proteins (OMPs), type 3 secretion systems T3SS, T2SS effectors, autoinducers, and iron-uptake proteins] have been targeted in clinical development, but to date, only three vaccines reached the phase III trials (Rello et al., 2017). The investigational vaccine IC43, a recombinant OMPs (OprF porin/OprI lipoprotein)–based vaccine, appeared to be the last promising based on the favorable safety and immunogenicity profile from phase II study (Adlbrecht et al., 2020).

### But What Is New in the Clinical Development Pipeline Since the 2017 WHO Alert?

Results from a phase II/III, multicenter, randomized, placebo-controlled, and double-blinded confirmatory study of IC43 vaccine against *P. aeruginosa* were published in 2020 (Adlbrecht et al., 2020). A total of 799 patients requiring mechanical ventilation received the vaccine at the time of admission to the intensive care unit (ICU). Although the study confirmed the safety profile and immunogenicity, the primary endpoint was not meet with the IC43 vaccine, providing no clinical benefit over placebo in terms of overall mortality (29.2% versus 27.7% in the IC43 and placebo groups, respectively, at day 28; p = 0.67) (Adlbrecht et al., 2020). The authors suggest that prevention of *P. aeruginosa* infection with a vaccine at the time of ICU admission may be too late. Indeed, in general, the humoral immune response is obtained about 2 to 3 weeks after the primary injection of the vaccine, which does not allow time to obtain a significant effect. Moreover, efficacy of the humoral response could be affected in some bacterial infections that are not systemic. An impact on mortality is also very difficult to demonstrate in critically ill patients, so another primary endpoint such as *P. aeruginosa*–related events would have been a better option. Nevertheless, this study demonstrated the feasibility of vaccinating a large cohort of ventilated patients in ICU and inducing a specific immune response (Adlbrecht et al., 2020).

**Figure 4:**
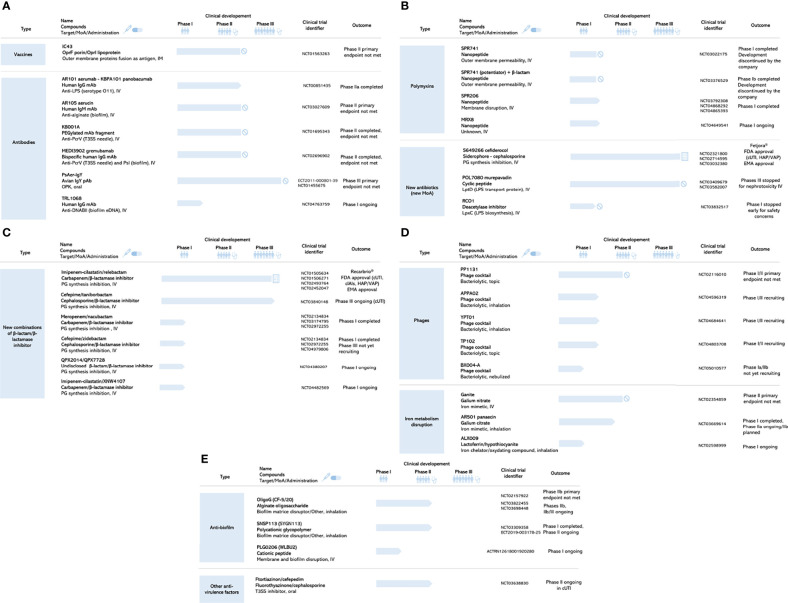
Search results: anti-Pseudomonas aeruginosa clinical development pipeline in December 2021. **(A)** Vaccines and antibodies. MoA, mode of action; IM, intramuscular; IV, intravenous; Ig; immunoglobulin; mAb, monoclonal antibody; pAb, polyclonal antibody; eDNA, extracellular desoxyribonucleic acid. **(B)** Polymixins and new antibiotics (new MoA). MoA, mode of action; IV, intravenous; PG, peptidoglycan; LPS, lipopolysaccharide. **(C)** New combinations of β-lactam/β-lactamase inhibitor. MoA, mode of action; IV, intravenous; PG, peptidoglycan. **(D)** Phages and Iron metabolism disruption. MoA, mode of action; IV, intravenous. **(E)** Anti-biofilm and other anti-virulence factors. MoA, mode of action; IV, intravenous; T3SS, type 3 secretion system.

On the basis of the literature search (Rello et al., 2017; Merakou et al., 2018; Bianconi et al., 2019; Adlbrecht et al., 2020; Sainz-Mejías et al., 2020), consultation of the clinical trials registry (ClinicalTrials.gov, 2021), and probably due to the recommendation published in the Cochrane Database of Systematic Reviews (Johansen and Gøtzsche, 2015), no vaccine is currently in clinical development (**Figure 4A**). An effective vaccine should be able to induce an humoral immune response capable of both mediating opsonophagocytic killing by phagocytic cells and neutralizing *P. aeruginosa* virulence factors (Sainz-Mejías et al., 2020). Advances have been made in understanding the interaction between *P. aeruginosa* and its host, and the key role of Th1 and Th17 host immune responses is well established and a cellular immune response mediated by Th17 cells (Sainz-Mejías et al., 2020). A deeper understanding of these mechanisms at each potential site and stage of infection would facilitate future vaccine development. A novel approach such as reverse vaccinology combined with genomic technologies has recently been described in *P. aeruginosa*, identifying potentially surface-exposed immunogenic proteins relevant in pathogenesis (Bianconi et al., 2019). The use of outer membrane vesicles, because it carries many surface antigens that can serve as targets, also represents a promising approach for vaccine development (Antonelli et al., 2021).

## Antibodies: An Immediate Passive Immunization to Treat or Prevent Infection

The vaccination (or active immunotherapy) strategy has various limitations, particularly for immunocompromised patients who may not be able to induce an appropriate immune response. Moreover, the immediate need for protection may not be met, including the time required for the development of an adequate immune response after vaccination, as discussed above (Adlbrecht et al., 2020). The uncommon mucus produced in the CF lung can also be a barrier to the systemic and specific immune response against *P. aeruginosa* that colonize the lower airways. Nevertheless, antibodies represent an effective and specific line of defense of the immune system. Passive immunotherapy with antibacterial monoclonal antibodies (mAbs) is therefore an alternative therapy against *P. aeruginosa* that could be interesting in certain clinical situations. Indeed, mAbs target specific surface antigens that are not usually the targets of antibiotics and are thus active against MDR bacteria. Antibacterial strategies are developed by mAbs binding to bacterial surface antigens, inducing opsonophagocytic killing by the host immune system or by binding to a virulence factor, such as toxin and thus neutralizing it (Lakemeyer et al., 2018). To date, no mAbs against *P. aeruginosa* have been approved.

Among the latest anti–*P. aeruginosa* mAbs, AR101 and AR105 have been developed as adjuvant strategy to antibiotics. Whereas AR101 targets the LPS serotype O11 surface O-antigen, AR105 targets alginate, a polysaccharide required for biofilm formation (Zurawski and McLendon, 2020; Lu et al., 2011). A phase IIa trial evaluating AR101 was completed in 2009 for hospital acquired pneumonia, showing promising results in clinical cure and survival rates (NCT00851435). A phase II trial testing the efficacy, safety, and pharmacokinetic of AR105 in addition to standard-of-care (SOC) antibiotics for pneumonia cause by *P. aeruginosa* was started in 2017 (NCT03027609). MEDI3902, a bispecific mAb, is able to recognize two different targets of *P. aeruginosa*, each containing a distinct epitope: the T3SS needle-tip protein PcrV, involved in host cell cytotoxicity and the surface exopolysaccharide Psl, involved in epithelial attachment and biofilm formation (**Figure 2**; Sato et al., 2011; Ryder et al., 2007). Designated as a Fast Track drug from Food and Drug Administration (FDA), MEDI3902 is under development for the prevention of healthcare-acquired *P. aeruginosa* pneumonia in high-risk patients^1^ . PsAer-IgY (egg yolk immunoglobulin), an avian polyclonal anti–*P. aeruginosa* antibody, has entered in a phase III trial to test its efficacy in preventing recurrence of *P. aeruginosa* infection in patients with CF (Thomsen et al., 2016).

### But What Is New in the Clinical Development Pipeline Since the 2017 WHO Alert?

Currently, in clinical development in China, the future of AR101 is unclear. Being specific to just one set of *P. aeruginosa* strains, AR101 mAbs may have limited value in contrast to MEDI3902 directed against independent and highly conserved target serotypes among clinical isolates (Tabor et al., 2018). In 2019, the developers reported that AR105 was unable to demonstrate superiority over SOC for clinical cure of *P. aeruginosa* pneumonia in phase II triala^2^ ; no further development resources were allocated to AR105. In 2018, several years after the completion of the phase II study in patients with CF infected with *P. aeruginosa*, results of KB001A were finally published (VanDevanter and Chmiel, 2018). KB001A is a monospecific PEGylated anti-PcrV mAb. Because no statistical difference was observed in time to antibiotic use compared to placebo, the primary endpoint was unfortunately not met. According to the authors, the low levels of T3SS secretion activity of *P. aeruginosa* isolates from patients with CF may be an explanation for the lack of efficacy observed with KB001A in this study (VanDevanter and Chmiel, 2018).

Early use in healthier patients may be effective in reducing the inflammatory effect responsible for lung disease progression (VanDevanter and Chmiel, 2018). Published in 2019, results from the phase I study of MEDI3902 in healthy subjects (Ali et al., 2019) supported further evaluation in phase II proof-of-concept study in mechanically ventilated ICU patients at high risk for *P. aeruginosa* pneumonia. Even when bispecific, MEDI3902 did not achieve the primary efficacy endpoint of reducing *P. aeruginosa* pneumonia (Chastre et al., 2020). Nevertheless, positive exploratory results were observed in subjects with lower levels of baseline inflammatory biomarkers, a subpopulation that could be tested to benefit from MEDI3902 (Chastre et al., 2020). Although PsAer-IgY was safe in the population studied, efficacy could not be demonstrated with the trial design used, as stated in the final report (EU Clinical Trials Register, 2018). The time to first recurrence of *P. aeruginosa* in patient sputum was not significantly different between active treatment and placebo. One explanation for this result could be that the non-specific IgY used as a comparator is also effective in postponing infection but in a non-specific way (EU Clinical Trials Register, 2018). The low efficacy could also be due to use antibodies from another specie in humans responsible of neutralizing anti-IgY antibodies production. TRL1068, a human mAb targeting DNA binding protein II (DNABII) is a promising new agent in clinical development (**Figure 4A**). By stabilizing extracellular DNA, DNABII is a key structural component of biofilm (Thi et al., 2020). Currently being tested in a phase I trial for prosthetic joint infection, TRL1068 is expected to eliminate the pathogen-protecting biofilm, making them more susceptible to antibiotic treatment (NCT04763759). The targeted epitope is highly conserved making the antibody potentially effective against biofilm produced by a wide range of clinically relevant pathogens, including 10 of the 12 WHO priority pathogens^3^ . Nevertheless, this efficacy could be low with early isolates that does not produce biofilm or are lower producers.

## Polymyxin Derivatives: A New “Old” Class of Antibiotics

Almost 60 years after their clinical approval, polymyxins remain a class of antibiotic available for many MDR Gram-negative bacteria but used as last-line therapeutic option due to their potential human nephro-and neurotoxicity (Li et al., 2019). Polymyxins are small cyclic cationic lipopeptides, interacting with the anionic lipid A component of LPS, in the outer membrane of Gram-negative bacteria, leading to cytoplasmic membrane disruption and bacterial cytotoxicity (Li et al., 2019). Their clinical use has restarted in recent years with polymyxin B and polymyxin E (colistin). On the basis of structure–activity–toxicity relationships, many efforts have been made to modify the original polymyxins and improve their safety profile. SPR741 is a polymyxin B derivative with less nephrotoxicity. SPR741 has no direct antibacterial activity but potentiates the efficacy of co-administered antibiotics (French et al., 2020), which alone would not have access to their intracellular targets (Corbett et al., 2017). SPR741 completed a phase I clinical trial in 2017 (Eckburg et al., 2019).

### But What Is New in the Clinical Development Pipeline Since the 2017 WHO Alert?

SPR741, combined with three different wide spectrum β-lactam antibiotics (ceftazidime, piperacillin/tazobactam, and aztreonam), showed favorable tolerability and PK profiles in a phase Ib study (Eckburg et al., 2019). The most effective combinations of such a “potentiator” strategy are not known yet, especially in non-healthy patient (Theuretzbacher et al., 2020). The clinical programs for SPR741 have been halted by the developers in 2020 before phase II in favor of the potentially more promising SPR206 molecule (WHO, 2021). This kind of strategic choice is also a reality of clinical development, explaining the dynamic change in the pipeline of molecules. SPR206 is another polymyxin B derivative, designed for use alone, and has shown antibiotic activity against MDR Gram-negative pathogens and XDR bacterial strains, including CRPA, in preclinical studies. It has thus a potential broad-spectrum of activity (Brown et al., 2019). Data from a phase I clinical trial were recently published with no evidence of nephrotoxicity and supporting further development of SPR206 (Bruss et al., 2021). Two additional phase I trials (**Figure 4B**) were also completed in December 2021: a bronchoalveolar lavage clinical trial assessing SPR206 penetration into the lungs, and a renal failure study. How polymyxin resistance and cross-​resistance to other classes might develop over time is not known. The benefit of SPR206 on polymyxin-​resistant strains must be demonstrated and lower toxicity must be shown in patients (Theuretzbacher et al., 2020). Nevertheless, the FDA has granted SPR206 Qualified Infectious Disease Product designation for the treatment of complicated urinary tract infections (cUTIs), hospital-acquired pneumonia (HAP), and ventilator-associated pneumonia (VAP) (Bruss et al., 2021), making this drug eligible for fast-track evaluation. MRX8, another polymyxin B derivative, started its clinical development on November 2020 (NCT04649541). MRX-8 was developed using a “soft drug design”, which represents a new approach to designing safer drugs by integrating metabolism and detoxification factors into the drug design process (Lepak et al., 2020).

## Antibiotics With a New Mode of Action for MDR Gram-Negative Bacteria

Among bacterial targets, peptidoglycan (PG) synthesis remains a privileged target with investigations on molecules of the β-lactams family. One field of development focuses on the modification active β-lactams by addition of an iron-chelating molecule, facilitating transport into bacteria (De Carvalho and Fernandes, 2014). Cefiderocol, a first in class of siderophore-cephalosporins, is able to bind to extracellular free iron, allowing active transport into the periplasmic space of Gram-negative bacteria through siderophore uptake systems. Cefiderocol subsequently binds to penicillin-binding proteins (PBPs), inhibiting bacterial PG cell wall synthesis that leads to cell lysis and death (EMA, 2020a). Although siderophore antibiotics have been investigated for several decades, none of them progressed to clinical development because of poor correlation between *in vitro* activity and *in vivo* efficacy or because of toxicity (El-Lababidi and Rizk, 2020). Promising assessment of cefiderocol for the treatment of cUTI in patients at risk of MDR Gram-negative infections started in phase II trial in 2015 (Portsmouth et al., 2018).

LPS that constitutes the OM outer layer at the surface of Gram-negative bacteria represents also an attractive target against pathogens (MacNair et al., 2020). By binding to the LPS transport protein D (LptD), the small cyclic peptide murepavadin causes a specific *P. aeruginosa* LPS biogenesis alteration (Martin-Loeches et al., 2018). A second phase II study of murepavadin co-administered with SOC in VAP due to *P. aeruginosa* finished in 2017 (NCT02096328).

### But What Is New in the Development Pipeline Since the 2017 WHO Alert?

In 2018, phase II results of cefiderocol versus imipenem-cilastatin, an available therapy for the treatment of cUTI were published. Response rates for the composite endpoint of microbiological eradication and clinical response were significantly higher in the cefiderocol arm compared with imipenem-cilastatin arm establishing the non-inferiority of cefiderocol (Portsmouth et al., 2018). The safety profile of cefiderocol in this study was good. On the basis of these results, cefiderocol was given priority review and was the first siderophore-antibiotic approved by the FDA in 2019^4^ , providing a new option of treatment of cUTI including pyelonephritis in patient with limited or no alternative treatment (FDA, 2019). Evaluated as non-inferior to meropenem for the primary endpoint of all-cause mortality in a phase III trial, cefiderocol has obtained an expanded indication for HAP and VAP (FDA, 2020; Wunderink et al., 2021). However, an increase in all-cause mortality death and infection-related death with treatment failure was observed in patients treated with cefiderocol compared with best available therapy in a descriptive phase III trial in critically ill patients with Gram-negative CR bacterial infections (Bassetti et al., 2021). The cause of the increase in mortality has not been established but close monitoring of the clinical response to therapy in patients with cUTI and HAP/VAP was asking by FDA (FDA, 2020). In 2020, the Committee for Medicinal Products for Human Use (CHMP) adopted a positive opinion, recommending the granting of a marketing authorization in Europe. The overall non-clinical and clinical data support the ability of cefiderocol to address an unmet need. The balance of benefits and risks is considered positive (EMA, 2020b).

Murepavadin fulfilled innovation criteria (WHO, 2020), including the main criteria for absence of known cross-resistance (Burrows, 2018), and reached phase III trials in HAP/VAP caused by *P. aeruginosa* (NCT03582007 and NCT03409679). Unfortunately, murepavadin was prematurely withdrawn from the authorization race on July 2019 due to high incidence of kidney injury^5^ , another hard reality of clinical development that can be interrupted even in late phase due to imbalance of benefit/risk ratio for patients. An alternative mode of administration of murepavadin, such as inhalation, is being explored to improve nephrotoxicity. A new phase I has been announced to start in 2022^6^ . In 2019, RC01, another new antibiotic targeting LPS biogenesis, entered in clinical development (**Figure 4B**). RC01 inhibits the bacterial enzyme LpxC, a key protein involved in the production of LPS lipid A. Despite demonstrated efficacy in clinical isolates of *P. aeruginosa* from patients with CF and good safety in preclinical phases, the phase I prematurely stopped for safety concerns (NCT03832517). As illustrated by the two previous antibiotics with new modes of action, murepavadin and RC01, novel targets or chemicals represent an unpredictable toxicity risk, as the transposition of safety signals from preclinical models to human is uncertain (Theuretzbacher et al., 2020).

## New Combinations of β-LACTAM/ β-LACTAMASE INHIBITOR A Strategy to Get Around Resistance

Among antibiotics, β-lactam targets specifically the PG biosynthesis through covalent binding/interaction to PBPs, the enzyme involved in late stages of PG synthesis. However, bacteria under selection pressure could produce β-lactamases, enzymes who hydrolyzed the β-Lactam ring. Hydrolyzing β-lactam antibiotics has made many of them ineffective becoming a major resistance issue in Gram-negative bacteria (Tümmler, 2019). Consequently, a synergic combination of β-lactam and an appropriate β-lactamase inhibitor (BLI) restoring β-lactam activity is a frequently used strategy. The well-known combination amoxicillin (β-lactam)/clavulanic acid (BLI) is a good example as it represents one of the most prescribed antibiotic worldwide and is a WHO-designed “core access antibiotic” that should be consistently available (Sharland et al., 2018).

Relebactam is a new BLI with activity against a broad range of β-lactamase enzymes including carbapenemases (Smith et al., 2020). *In vitro* addition of relebactam to imipenem restored imipenem activity against several imipenem-resistant bacteria, including *P. aeruginosa* (Smith et al., 2020). Relebactam in association with imipenem-cilastatin demonstrated efficacy in phase II trials dedicated to cUTIs (Sims et al., 2017) and complicated intraabdominal infections (cIAIs) (Lucasti et al., 2016), thus entered in phase III trial in HAP/VAP (Titov et al., 2020) versus piperacillin-tazobactam– and imipenem-resistant pathogens versus colistin (Motsch et al., 2020), a polymixin used as last resort therapy. Taniborbactam is a highly potent pan-spectrum new BLI that inhibits all classes of β-lactamase enzymes (Liu et al., 2020). In combination with the fourth-generation cephalosporin cefepime, taniborbactam positively completed the phase I milestone in 2017 and thus supported other trials (Dowell et al., 2020).

### But What Is New in the Clinical Development Pipeline Since the 2017 WHO Alert?

Evaluation of phases II/III clinical data (Lucasti et al., 2016; Sims et al., 2017; Motsch et al., 2020; Titov et al., 2020) demonstrates that the relebactam/imipenem-cilastatin association is well tolerated and effective in the treatment of cUTIs, cIAIs, and HAP/VAP and obtained FDA approval for these therapeutic indications^7^ ^,^ ^8^ . Taniborbactam/cefepime association demonstrated *in vitro* rescue of cefepime activity by taniborbactam against clinical isolates of CRPA (Hamrick et al., 2020). As the FDA designated Fast Track, the association skipped phase II and is currently on phase III trial to access the safety and efficacy compared with meropenem in both eradication of bacteria and in symptomatic response as primary endpoint in patients with cUTIs including acute pyelonephritis (NCT03840148). Another phase III trial in patients with HAP/VAP is scheduled to begin in 2022^9^ . At least four other new BLIs in association with β-lactam are on phase I stage (completed or ongoing) (**Figure 4C**): nacubactam, zidebactam, QPX7728, and XNW4107. Whereas nacubactam reported narrow *in vitro* activity in *P. aeruginosa* (Asempa et al., 2020), QPX7728 is an ultra-broad-spectrum β-lactamase inhibitor with the broadest spectrum of inhibition *in vitro* reported to date in a single BLI molecule (Lomovskaya et al., 2021). Zidebactam in association with cefepime should start a phase III in cUTI or acute pyelonephritis end of 2021 (NCT04979806). Despite this antibiotic-based strategy, other mechanisms can unfortunately confer resistance to β-lactam/BLI new combinations. Beyond β-lactamases production, overproduction or extended-spectrum β-lactamases, *P. aeruginosa* has developed efficient mechanisms as decreased permeability of the outer membrane and overproduction of efflux pumps (Moradali et al., 2017).

## Phage Therapy: Using a Bacteria Natural Killer

Bacteriophages that infect and lyse their target bacteria are being reconsidered as an alternative therapy to treat MDR bacterial infections. Interestingly, some phages are also able to disrupt biofilm barrier with EPS-depolymerase activity (Cornelissen et al., 2012). Phage therapy includes mono-phage, phages-cocktail, phage-derived enzyme (lysin), bio-engineered phage, and phage combined with antibiotics (Patil et al., 2021). Systematic literature search shows a large number of case study reports and compassionate use for severe patients in specialized centers but despite promising results recent clinical trial evidence-based will be necessary. The first randomized controlled trial using a cocktail of natural lytic *P. aeruginosa* phages for the topical treatment of infected burn wound patients was stopped on January 2017 because of PP1131 insufficient efficacy versus SOC (Jault et al., 2019).

### But What Is New in the Clinical Development Pipeline Since the 2017 WHO Alert?

In 2019, full results of the phase I/II trial testing PP1131 were published. This study had several limitations and encountered many unexpected difficulties. An ancillary analysis showed that the bacteria isolated from patients with failed PP1131 treatment were resistant to low phage doses (Jault et al., 2019). Phage cocktails of predefined composition could negatively interfere in the relations between phage and bacterium by selecting phage resistance in the bacterial populations that vary among patients (Tümmler, 2019). An individually approach could be a more effective using phagograms to study *in vitro* the sensitivity of *P. aeruginosa* to bacteriophages in the manner of antibiograms, and a phage collection for personalized treatment (Friman et al., 2016).

APPA02^10^ , a new inhaled cocktail of complementary phages, is currently tested for safety and tolerability in a phase I/II trial to treat serious respiratory infections, with an initial emphasis on patients with CF (NCT04596319). *P. aeruginosa* recovery in sputum following multiple doses of treatment will be also explored. In March 2021, a phase I/II trial was initiated for assessment of another targeted inhaled phage therapy YPT01^11^ added to standard antimicrobial therapy in the treatment of chronic *P. aeruginosa *infections in CF (NCT04684641). A multispecies targeting topic phage cocktail TP102^12^ was formulated against *P. aeruginosa* but also *Staphylococcus aureus *and *Acinetobacter baumannii*. TP102 started a safety evaluation in subjects with both non-infected and infected diabetic foot ulcers as the primary endpoint and targeted bacteria clearance as secondary endpoint (NCT04803708). Results of these three early phase studies for APPA02, YPT01, and TP102 assessment are expected in 2022. BX004-A, a nebulized bacteriophage therapy, entered in a phase Ia/IIb (**Figure 4D**). Exploratory objectives include whether this treatment reduces sputum *P. aeruginosa* bacterial load in CF subjects with chronic *P. aeruginosa* pulmonary infection (NCT05010577). During the same time, other new topical phage therapies by spray as an adjunct to SOC therapy for the prevention and treatment of *P. aeruginosa*, *S. aureus*, or *Klebsiella pneumoniae* infections will enter in development in pressure ulcers (PL03BM, NCT04815798) and burns (PGX0100, NCT04323475). Despite promising results, both historically and recently, the efficacy of phage therapy has still not been sufficiently examined and documented in high-quality clinical trial in humans to answer the questions raised about how to best use bacteriophages (Leitner et al., 2021).

## Depriving Bacteria From a Survival Essential Element: Iron

As an essential nutrient for growth and biofilm establishment, *P. aeruginosa* has developed different strategies to acquire iron from its environment (haem or siderophores mediated uptake systems and porins) (Zhang et al., 2021). Host fights infection reducing iron biodisponibility for its invading bacteria using, for example, transferrin in serum and lactoferrin in mucosal secretion (Zhang et al., 2021). Acting as an iron mimetic, gallium disrupts multiple iron-dependent synthetic and metabolic pathways (Frei, 2020). Preliminary clinical study raised the possibility that gallium may be a safe and effective treatment for human infections (Goss et al., 2018). A phase II study to test IV gallium nitrate to control  *P.* *aeruginosa * infections in adults with CF started in 2016 (NCT02354859). Because the IV form of gallium involves a continuous 5-day infusion, which is a demanding treatment regimen for patients, the AR501 inhaled formulation of gallium citrate self-administered *via* a nebulizer device was designed to be given once a week, allowing for direct delivery to the lungs^10^. Another antimicrobial with an activity on iron metabolism is ALX-009, a combination of lactoferrin and hypothiocyanite, natural major antimicrobial substances deficient in the airway secretions of patients with CF (Tunney et al., 2018). Lactoferrin deprives bacteria of iron due to its iron chelator activity and can bind to cell membranes, leading to alterations in permeability and enhancing bacterial killing by other antibiotics (Rogan et al., 2004). Hypothiocyanite is a highly reactive compound that oxidizes proteins to created disulfide bonds that perturb the bacterial physiology (Thomas et al., 1978). ALX009 started a phase I in 2015 as an inhalable solution administered through nebulization (NCT02598999).

### But What Is New in the Clinical Development Pipeline Since the 2017 WHO Alert?

The phase II clinical trial in adults with CF found no significant difference between the number of responders (defined as a participant having a 5% or greater increase in lung function by day 28) in the IV gallium nitrate and placebo group^13^ . The primary endpoint was unmet, however a significantly reduction of *P. aeruginosa* was found in the sputum of gallium treated patients compared to the placebo group for participants who were culture positive for *P. aeruginosa* at baseline^14^ . Overall, IV gallium nitrate was well-tolerated compared to placebo. With broad-spectrum anti-infective activity AR501 is being developed in a phase I/IIa (**Figure 4D**) to treat chronic bacterial lung infections of patients with CF (NCT03669614). On 2020, positive safety data were reported from the phase I portion of the study, and AR-501 has been granted Fast Track, Qualified Infectious Disease Product, and Orphan Drug Designation by FDA and EMA. The originally planned protocol design was adapted into a phase IIa (dose selection/sample size determination) which results expected in 2022 will be implemented directly in a phase IIb study (efficacy) using the same clinical study protocol^15^ . This is an encouraging example of simplified and accelerated development supported by Health Authorities in a context of urgent need. Final data collection date for primary outcome measure of ALX009 in healthy volunteers and patients suffering from CF and non-CF bronchiectasis was expected in October 2021 (NCT02598999).

## Anti-Biofilm Strategy

Among virulence factors of *P. aeruginosa*, biofilms increase bacterial adherence, immune system evasion, and antibiotic tolerance by blocking the diffusion of positively charged drugs. OligoG is an alginate oligosaccharide with the potential to reduce sputum viscosity of patients with CF by chelating calcium (Ermund et al., 2017), easing clearance of mucus from patient airways, reducing microbial burden and inflammation. OligoG was also shown to disrupt biofilm structure of *P. aeruginosa* mucoid phenotype (Powell et al., 2018) and could in consequence improve host immune system action and the effectiveness of antimicrobial agents. To determine the safety and local tolerability of multiple dose administration of inhaled fragment in healthy volunteers, a phase I study was performed with a particular attention on pulmonary functioning and adverse events (NCT00970346).

### But What Is New in the Clinical Development Pipeline Since the 2017 WHO Alert?

In a first phase II study in adult CF subjects with *P. aeruginosa* lung infection, inhalation of OligoG powder over 28 days was shown to be safe; however, statistically significant improvement of FEV_1_ (forced expiratory volume by the patient in one second) was not reached (Van Koningsbruggen-Rietschel et al., 2020). Lung function is widely used as primary outcome measure in the development of drugs to treat CF but regarding its mode of action could not be adapted to prove an OligoG efficacy, especially when disease is already installed. *Post hoc* subgroup analyses support mechanism of action for OligoG and warrant further prospective studies (Van Koningsbruggen-Rietschel et al., 2020). OligoG has Orphan Drug designation from both the EMA and the FDA and is currently tested in other phase IIb studies in addition to SOC compared to placebo with SOC too (NCT03698448 and NCT03822455). SNSP113 (SYGN113), a novel large polycationic glycopolymer, poly (acetyl, arginyl) glucosamine, was tested in a phase I study in healthy subjects and patients with stable CF (NCT03309358) and is currently evaluated in a phase II study (**Figure 4E**). PLG0206 (WLBU2), an engineered cationic antimicrobial peptide with broad-spectrum activity and preventing *in vitro P. aeruginosa* biofilm growth on airway epithelial cells (Lashua et al., 2016), received Orphan Drug status for prosthetic joint infection and entered in phase 1 trial in healthy subjects in 2018. PLG0206 will enter a phase 1b for treatment of periprosthetic joint infection in 2022 (NCT05137314).

## Other Innovative Anti-Virulence Strategies

As T3SS and associated toxins are major virulence factors of *P. aeruginosa* (**Figure 2**), innovative therapeutic strategies are developed to reduce the infection severity. Despite many hopes based on *in vitro* or preclinical activities, only one treatment targeting T3SS is currently in clinical development (**Figure 4E**). Ftortiazinon, a small molecule with a strong potential as an antibacterial therapy (Sheremet et al., 2018), developed by the Gamaleya Research Institute, entered a phase 2 in combination with cefepime for the treatment of patients with cUTI caused by *P. aeruginosa* in 2018 (NCT03638830).

## Discussion

**Figure 5:**
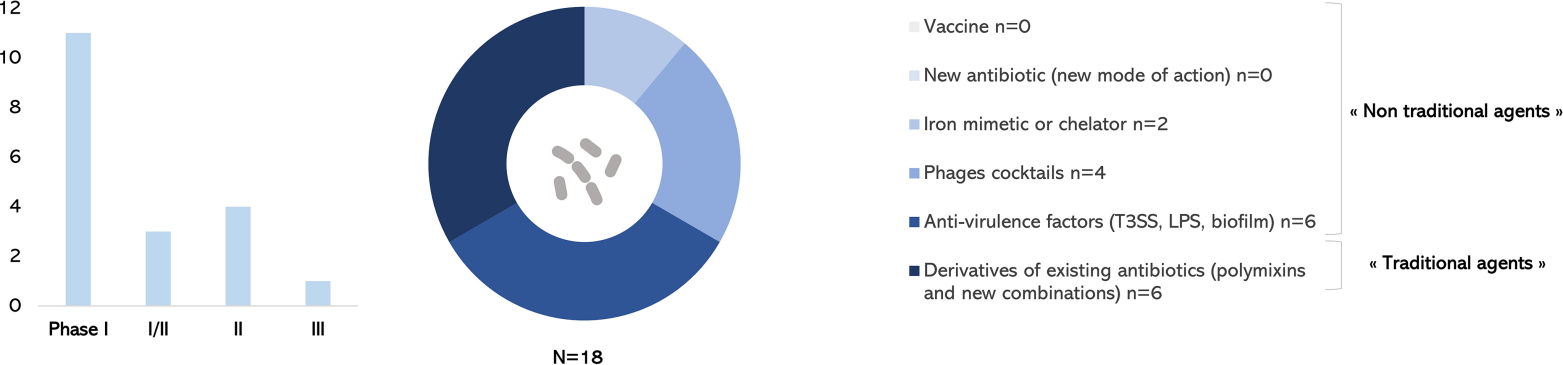
Anti–*Pseudomonas aeruginosa* treatments in clinical development in December 2021.

This review aimed at providing an up-to-date picture of therapeutics against *P. aeruginosa* currently in clinical development, since the 2017 WHO alert. Only one antibacterial drug with a new mode of action has been approved by FDA and EMA against *P. aeruginosa* (**Figure 4B**). Among 18 drugs of interest anti–*P. aeruginosa* in the development pipeline described in this review, only one new combination of β-lactam/β-lactamase inhibitor of antibiotics is in phase III trial (**Figure 4C**). Derivatives of existing antibiotics considered as “traditional agents” are highly represented (**Figure 5**). Diverse “non-traditional agents” including bacteriophages, iron mimetic/chelator, and anti-virulence factors are significantly represented but unfortunately still in early clinical stages. There is no vaccine in development to prevent *P. aeruginosa* infections despite a half century of research effort specifically focused on this challenge (Sainz-Mejías et al., 2020; ClinicalTrials.gov., 2021).

Studying pipeline anti–*P. aeruginosa* since 2017 up to now shows how development of a new treatment can be a difficult process. Lack of correspondence between *in vitro* or preclinical study and phase II clinical response questions the choice of pertinent animal models recreating human infection conditions (Theuretzbacher et al., 2019; Theuretzbacher et al., 2020). Methodology used is often a strategic issue, with the crucial definition of the clinically significant primary outcome (Merakou et al., 2018; Adlbrecht et al., 2020) and the infection-site that could best allow to meet efficacy criteria. The absence of statistically significative evidence is not the evidence of absence of efficacy. We can question, for example, if death is a good primary outcome when critically ill patients are entering in ICU (Merakou et al., 2018) and if lung function measure is a pertinent primary outcome for a CF patient with chronical severe disease (Ali et al., 2019; Van Koningsbruggen-Rietschel et al., 2020). Studying pipeline also underlines that a late clinical stage development can be interrupted due to unexpected toxicity^16^ or bankrupt of a biotechnology company. COVID-19 pandemic had also an important impact on clinical trials recruitment and timelines during the last 2 years.

*P. aeruginosa*–host interactions and host immunometabolism are not yet enough understood, complicating the development of effective therapies and vaccines (Sainz-Mejías et al., 2020). Because of the fact that *P. aeruginosa* is an opportunistic bacterium, patients infected with *P. aeruginosa* are mostly immunocompromised individuals. This makes it difficult to develop a vaccine for these patients (Yaeger et al., 2021). As *P. aeruginosa* is characterized by its genomic plasticity, drugs or vaccine must be designed to target conserved elements between strains to ensure an optimal efficacy (Tse et al., 2017). This constitutes a true limitation regarding strains like PA7, a non-respiratory MDR that lacks the T3SS and its effectors or the exotoxin A (Roy et al., 2010) or the Liverpool epidemic strain, a lineage with enhanced virulence and antimicrobial resistance characteristics (Salunkhe et al., 2005).

Each class of treatment against *P. aeruginosa* presents strengths, weaknesses, opportunities, and threats that have to be taken into account in global clinical development considerations (**Table 1**). Among “non-traditional agents”, a vaccine could offer a long-term sustainable approach to infection prevention because it will decrease the need for antibiotics and hence the emergence of antibioresistance (Micoli et al., 2021). Anti-virulence strategy may have the potential through novel, specialized, and non-killing modes of action to reduce the selective pressure responsible of MDR (Dickey et al., 2017) or select less virulent strains. Regarding combinatory multifactorial virulence, an anti-virulence factor used alone do not has therapeutic utility and hence must be used as pre-emptive or adjunctive treatment in combination with traditional antibiotics (Dickey et al., 2017). Despite many years of research, no anti-virulent agent has yet been introduced in clinical use against *P. aeruginosa*. Novel targets for anti-virulence strategy must be proposed and T6SS as a key virulence factor of *P. aeruginosa* (**Figure 2**) could be selected. Inhibition of T6SS assembly or neutralization of its toxins (amidases, phospholipases, protease, pore forming toxin, and iron-acquisition effector) (**Figure 2**) would allow to interfere at two moments of the infectious process: (i) during host colonization (competition for the niche with microbiota or other pathogens in case of CF patients) and (ii) escape from the immune system (internalization and autophagy).

**Table 1:** Strengths and weaknesses (internal factors), opportunities and threats (external factors) of each class of treatment in clinical development against *P. aeruginosa*.

Type	Strengths	Weaknesses	Opportunities	Threats
**Vaccines**	- Prophylactic strategy with a response in early stage of infection - Multitargeting possible/specificity - Reduced probability of resistance - Well define target population (high risks patients for opportunistic infection to improve immunity)	- Non-immediate action - Limited predictive value of animal models (immune system complexity) - Weak preclinical pipeline - No vaccine currently in clinical trial - Immunization dependent of the patient immune system status	- COVID-19 vaccine development change of paradigm - New technologies (reverse vaccinology, adjuvants optimization, mRNA) - Spread of MDR as a reason to consider vaccination	- Image of low morbidity/mortality of *P. aeruginosa* infection in general population - Burden of disease and incidence rate not well define in high-risk patients - Development mostly in health-care associated pneumonia - Difficulties to generate robust data to support approval (how to design clinical trial regarding complexity of infections) - Non-inferiority clinical trial (design strategy with lack of distinct benefit over existing treatment) - Non-MDR arm used in the studies design; difficulties to recruit patients with MDR - Duration of clinical trial in the current development paradigm - High-risk strategies for innovative treatment (new targets or new type of drug; high attrition rate of phase I) - Cost of diagnosis before use of drugs with narrow spectrum - Cost of biotherapies manufacturing *versus* traditional drugs - Strong dependence on public and/or philanthropic funding - High need of innovation not or partially covered - Lack of commercial interest in developing new antibacterial drugs (high risk development, low return on investment expected, new drugs will be used as last resort) - Low economic value of novel antibiotic *versus* innovative treatment of chronical diseases - Many big pharmaceuticals companies abandoned R&D programs - Challenge of clinical development by biotechnologies companies
**Antibodies**	- Immediate protection (preventive or adjunctive therapy possible) - Immunization independent of the patient immune system status - Multitargeting possible/specificity - Anti-virulence factors strategy with probability of reduced resistance - Narrow spectrum avoiding the disruption of microbiota	- Mostly intravenous administration not ideal for immunocompromised patients - Large proteins - Usually narrow spectrum of activity necessitating diagnosis before to treat (specialized and costly health-care facilities)	- mAb technology well known in cancer or autoimmune diseases treatment - Manufacturing methods and safety profile well established - DNA mAb to overcome cost	
**Polymyxins**	- Broad-spectrum activity - Potentiate and extend the spectrum of conventional antibiotics (synergy) - Efficacy against both quiescent and growing bacteria	- Emergence of resistance - Large spectrum of activity engendering dysbiose - Possible toxicity against host - Currently last line of defense	- No newer alternatives: the urgent need to optimize their clinical use - Substantial progress made in understanding complexity of polymyxins and “soft drug design”	
**New antibiotics** **(new MoA)**	- New mode of action less susceptible to induce resistance - Broad or narrow activity spectrum	- Based on low evidence, clinicians appear reluctant to use new antibiotic agents - Safety profile less known	- Substantial knowledge of rich ecological niches that produces antibiotics as secondary metabolite - Human microbiota research enthusiasm	
**New combinations of β-lactam/β-lactamase inhibitor**	- Synergic effect, restoring activity of β-lactam - Counteract β-lactamase defense strategy	- Resistance mechanisms beyond the production of β-lactamases - Broad-spectrum of antibiotic resistance/cross resistance - Short-term option	- Highly developed antibacterial β-lactam based clinical pipeline.	
**Phages**	- Self amplification at infection site - Biofilm penetration (possible lysis) - Specificity of action avoiding microbiome disruption - Escape mutants could be less pathogenic due to loss of surface factors expression	- Lack of knowledge about phage mode of action - Strong selective pressure to develop resistance - Diagnosis necessary for personalized therapy - Immunogenicity of phage	- Availability for patients in Eastern Europe specialized centers - Compassionate use as clinical experience - Cost effective - Human microbiome research (including largely phagome)	
**Iron metabolism disruption**	- Activity against Gram-negative and Gram-positive (broad spectrum of activity)	- Production of high level of siderophore pyoverdine to compensate - Lack of knowledge about exact mode of action	- Untapped potential of metal-based antibiotics versus organics compounds	
**Anti-biofilm**	- Sensibilize bacteria to antibiotic - Strategy with reduced probability of resistance - Can supplement antibiotics for increase efficacy - Specificity of action avoiding microbiota depletion	- Requires a combination therapy - Effective in strain infection with mucoid phenotype	- Substantial knowledge of virulence mechanisms of pathogen bacteria - Biofilm well recognized as a threat in healthcare institutions	
**Other anti-virulence factors**	- Strategy with reduced probability of resistance or selection of less virulent strains - Specificity of action avoiding microbiota depletion	- Diagnosis necessary for personalized therapy - Plasticity of virulence factors expression - Require a combination therapy	- The rise of anti-virulence strategy (large number of putative virulence targets) - Anti-virulence drugs already approved (exotoxins blockage)	

This table is based on the following references for vaccines (Merakou et al., 2018; Bianconi et al., 2019; Theuretzbacher et al., 2020; Sainz-Mejías et al., 2020; Micoli et al., 2021; Antonelli et al., 2021), antibodies (Lakemeyer et al., 2018; Theuretzbacher et al., 2020; Adlbrecht et al., 2020; Yaeger et al., 2021; Zurawski and McLendon, 2020), polymyxins (Li, et al., 2019; Theuretzbacher et al., 2020; Lepak et al., 2020), new antibiotics (WHO, 2021; Dickey et al., 2017; Tse et al., 2017), new combinations of β-lactam/β-lactamase inhibitor (WHO, 2021; Theuretzbacher et al., 2020), phages (Friman et al., 2016; Jault et al., 2019; Patil et al., 2021), iron metabolism disruption (Zhang et al., 2021; Frei et al., 2020), anti-biofilm (Dickey et al., 2017), and other anti-virulence factors (Dickey et al., 2017; Theuretzbacher et al., 2020).

Current antibiotics are less effective due to increasing resistance, and some *P. aeruginosa* isolates are resistant to all available treatments, underlying the unmet medical need. Given the development duration, the pipeline remains unsatisfactory leading best case to the approval of new antibacterial drugs that treat CRPA in several years. In addition, as the United Nations, WHO, and numerous experts published in an April 29, 2019, report, immediate, coordinated, and ambitious action must be taken to avoid a potentially disastrous antimicrobial resistance crisis. If not, drug-resistant diseases could cause 10 million deaths each year by 2050 warns the UN Interagency Coordination Task Force on Antimicrobial Resistance^16^. Developing a new treatment takes years, however, COVID-19 pandemic has demonstrated that it is possible to accelerate the development of a molecule or a vaccine in case of crisis (Krammer, 2020). Beyond the investments needed to build a robust pipeline, the Community needs to reinvent medicine with new strategies of development to avoid the disaster.

## Author Contributions

SR, AG, and SB wrote the manuscript. All authors contributed to the article and approved the submitted version.

## Funding

Work of SB team is funded by the Centre National de la Recherche Scientifique, the Aix-Marseille Université, and by grant from the Agence Nationale de la Recherche (ANR-21-CE11-0028-01). AG is supported by “Vaincrela mucoviscidose” (VLM) and “Association Grégory Lemarchal”(grant numbers RF20190502416 and RF20210502854), ANR-15-IDEX-02, SATT Linksium, and Fondation Université Grenoble Alpes. SATT Linksium was not involved in the study design, collection, analysis, interpretation of data, the writing of this article or the decision to submit it for publication.

## Conflict of Interest

The authors declare that the research was conducted in the absence of any commercial or financial relationships that could be construed as a potential conflict of interest. SR is currently working in the Medical Department of Novartis Gene Therapies France SAS as Senior Medical Science Manager. SR is a PhD student independently of his professional position and on a totally different therapeutic area.

## Publisher’s Note

All claims expressed in this article are solely those of the authors and do not necessarily represent those of their affiliated organizations, or those of the publisher, the editors and the reviewers. Any product that may be evaluated in this article, or claim that may be made by its manufacturer, is not guaranteed or endorsed by the publisher.

## Footnotes

1
AstraZeneca website: https://www.astrazeneca.com/media-centre/press-releases/2014/medimmune-fda-medi3902-nosocomial-pneumonia-prevention-23092014.html#modal-historic-confirmation [Accessed December 2021]

2
Aridis Pharmaceuticals website: https://investors.aridispharma.com/2019-09-03-Aridis-Pharmaceuticals-Reports-Phase-2-Clinical-Trial-Results-of-AR-105-for-the-Treatment-of-Ventilator-Associated-Pneumonia-Caused-by-Pseudomonas-Aeruginosa [Accessed December 2021].

3
Trellis bioscience website: www.trellisbio.com/pipeline/bacteria.html [Accessed December 2021].

4
FDA News Release December 16, 2019. https://www.fda.gov/news-events/press-announcements/fda-approves-new-antibacterial-drug-treat-complicated-urinary-tract-infections-part-ongoing-efforts [Accessed December 2021].

5
Polyphor website: https://www.polyphor.com/news/corporate-news-details/?newsid=1800485[Accessed December2021].

6
Polyphor website: https://www.polyphor.com/news-adhoc/news-detail/?newsid=2176875 [Accessed December2021].

7
FDA website: FDA approves new treatment for complicated urinary tract and complicated intra-abdominal infections | FDA [accessed December 2021].

8
FDA website: FDA Approves Antibiotic to Treat Hospital-Acquired Bacterial Pneumonia and Ventilator-Associated Bacterial Pneumonia | FDA [Accessed December 2021].

9
Venatorx website: https://www.venatorx.com/press-releases/venatorx-pharmaceuticals-provides-update-on-cefepime-taniborbactam/ [Accessed December 2021].

10
Armata Pharmaceutical website : https://www.armatapharma.com/pipeline/ap-pa02/ [Accessed December 2021].

11
Felix Biotechnology website: https://www.felixbt.com/new-page [Accessed December 2021].

12
Technophage website: http://technophage.pt/pipeline/ [Accessed December 2021].

13
Aridis pharmaceutical website: https://www.aridispharma.com/ar-501/ [Accessed December 2021].

14 Cystic Fibrosis Foundation website : https://www.cff.org/Trials/Finder/details/374/IGNITE-Safety-and-effectiveness-of-gallium-nitrate-in-adults-with-cystic-fibrosis [Accessed December 2021].

15
Aridis Pharmaceuticals website: Aridis Announces Agreement with the FDA on Updated Phase 2 Clinical Trial Design for AR-501 - Sep 8, 2020 (aridispharma.com) [Accessed December 2021].

16 WHO website news release on 29 April 2019: https://New report calls for urgent action to avert antimicrobial resistance crisis (who.int) [Accessed December 2021]
